# Redetermination of Mg_2_B_25_ based on single-crystal X-ray data

**DOI:** 10.1107/S1600536812023768

**Published:** 2012-05-31

**Authors:** Luca Bindi, Alessandro Figini Albisetti, Giovanni Giunchi, Luciana Malpezzi, Norberto Masciocchi

**Affiliations:** a Dipartimento di Scienze della Terra, University of Firenze, Via La Pira 4, I-50121 Firenze, Italy; b Edison Spa R&D, Foro Buonaparte 31, I-20121 Milano, Italy; c Dipartimento di Chimica, Materiali, Ingegneria Chimica "G. Natta", Politecnico di Milano, Via Mancinelli 7, I-20131 Milano, Italy; d Dipartimento di Scienze Chimiche e Ambientali, University of Insubria, Via Valleggio 11, I-22100 Como, Italy

## Abstract

The crystal structure of Mg_2_B_25_, dimagnesium penta­eicosa­boride, was reexamined from single-crystal X-ray diffraction data. The structural model previously reported on the basis of powder X-ray diffraction data [Giunchi *et al.* (2006 ▶). *Solid State Sci.* **8**, 1202–1208] has been confirmed, although a much higher precision refinement was achieved, leading to much smaller standard uncertainties on bond lengths and refined occupancy factors. Moreover, all atoms were refined with anisotropic displacement parameters. Mg_2_B_25_ crystallizes in the β-boron structure type and is isostructural with other rhombohedral compounds of the boron-rich metal boride family. Magnesium atoms are found in inter­stitial sites on special positions (two with site symmetry .*m*, one with .2 and one with 3*m*), all with partial occupancies.

## Related literature

For background to this class of compounds, see: Nagamatsu *et al.* (2001 ▶); Giunchi (2003 ▶), Giunchi *et al.* (2006 ▶). For related phases in the Mg–B system, see: Adasch *et al.* (2006**a* ▶,b* ▶); Brutti *et al.* (2002 ▶); Guette *et al.* (1981 ▶); Jones & Marsh (1954 ▶); Naslain *et al.* (1973 ▶); Russell *et al.* (1953 ▶); Pediaditakis *et al.* (2010 ▶).

## Experimental

### 

#### Crystal data

Mg_2_B_25_
*M* *_r_* = 318.87
Hexagonal, 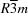

*a* = 11.0398 (5) Å
*c* = 24.195 (1) Å
*V* = 2553.75 (19) Å^3^
*Z* = 12
Mo *K*α radiation
μ = 0.23 mm^−1^
*T* = 298 K
0.08 × 0.07 × 0.07 mm

#### Data collection

Oxford Xcalibur 3 CCD diffractometer
Absorption correction: gaussian (*ABSPACK*; Oxford Diffraction, 2006 ▶) *T*_min_ = 0.982, *T*_max_ = 0.984
4794 measured reflections
799 independent reflections
724 reflections with *I* > 2σ(*I*)
*R*_int_ = 0.045

#### Refinement

*R*[*F* ^2^ > 2σ(*F* ^2^)] = 0.021
*wR*(*F* ^2^) = 0.049
*S* = 1.13
799 reflections
122 parameters
Δρ_max_ = 0.14 e Å^−3^
Δρ_min_ = −0.31 e Å^−3^

### 

Data collection: *CrysAlis CCD* (Oxford Diffraction, 2006 ▶); cell refinement: *CrysAlis CCD*; data reduction: *CrysAlis RED* (Oxford Diffraction, 2006 ▶); program(s) used to solve structure: *SHELXS97* (Sheldrick, 2008 ▶); program(s) used to refine structure: *SHELXL97* (Sheldrick, 2008 ▶); molecular graphics: *DIAMOND* (Brandenburg, 2001 ▶); software used to prepare material for publication: *SHELXL97*.

## Supplementary Material

Crystal structure: contains datablock(s) global, I. DOI: 10.1107/S1600536812023768/wm2631sup1.cif

Structure factors: contains datablock(s) I. DOI: 10.1107/S1600536812023768/wm2631Isup2.hkl

Additional supplementary materials:  crystallographic information; 3D view; checkCIF report

## Figures and Tables

**Fig. 1.**
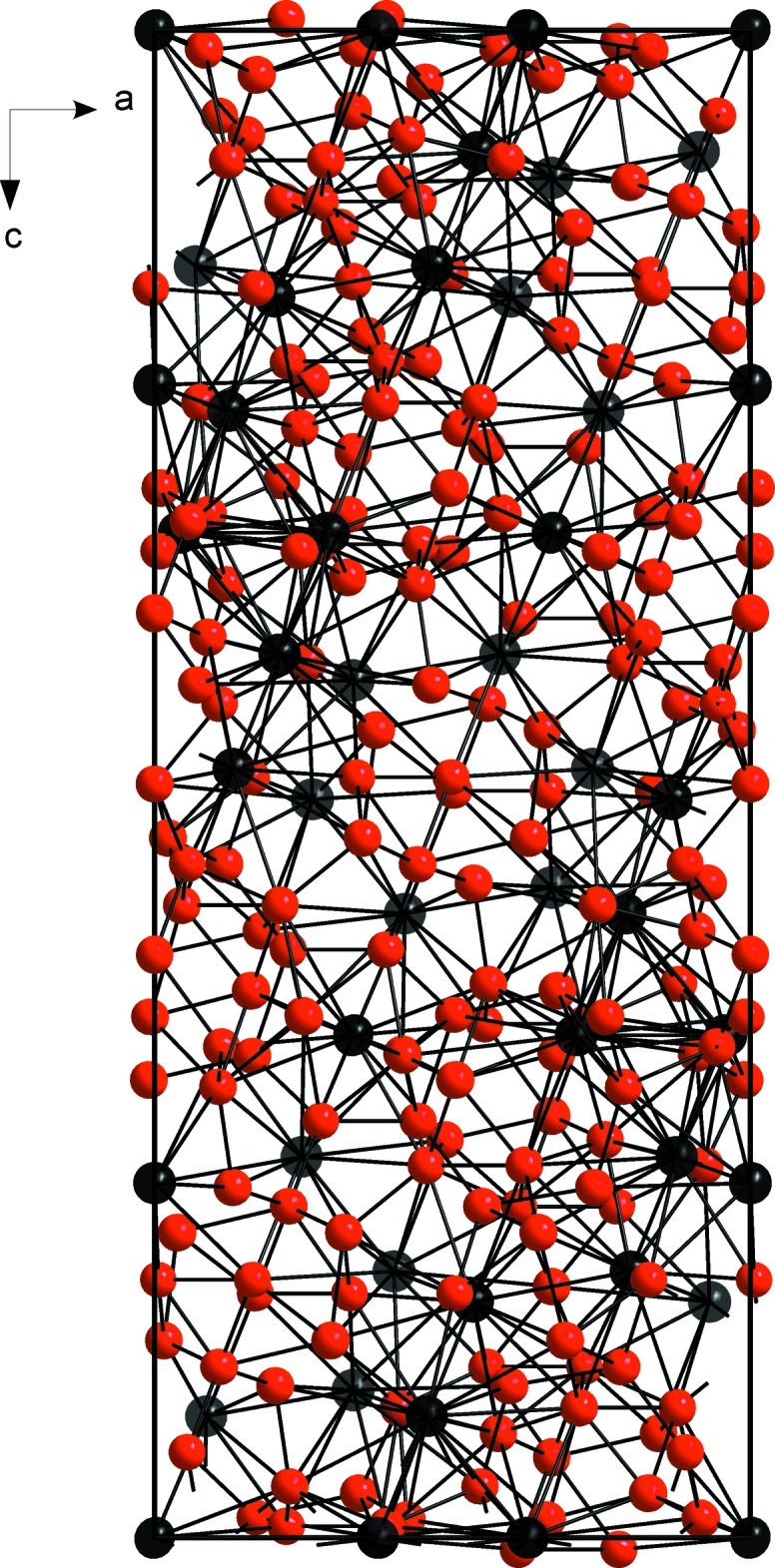
The crystal structure of Mg2B25 viewed down [010]. Anisotropic displacement parameters are drawn at the 90% probability level. Mg atoms are black, B atoms red.

## supplementary crystallographic information

### Comment

The interest for a detailed description of the phase diagram of the Mg–B system has greatly increased with the recent discovery of the superconductivity of the MgB_2_ species (Nagamatsu *et al.*, 2001). Up to now the only well-characterized crystalline phases of this binary system are the MgB_2_ (Russell *et al.*, 1953; Jones & Marsh, 1954), MgB_4_ (Naslain *et al.*, 1973), MgB_7_ (Guette *et al.*, 1981), MgB_12_ (Adasch *et al.*, 2006*a*), MgB_17.4_ (Adasch *et al.*, 2006*b*), MgB_20_ (Brutti *et al.*, 2002) and Mg_5_B_44_ (Pediaditakis *et al.*, 2010) species. Giunchi *et al.* (2006) reported on the crystal structure determination, by laboratory X-ray powder diffraction (XRPD) methods, of the Mg_2_B_25_ species. This compound was isolated during a side study of the research aimed at the synthesis of MgB_2_ by infiltration of crystalline boron with liquid Mg (Giunchi, 2003) (process identified by Mg-RLI: Mg-Reactive Liquid Infiltration) in reaction conditions very different from the previous *R*3*m* members of the same family, *i.e.* with temperatures lower than 1033 K and reaction times of the order of one hour, in contrast to 1873 K and 40 h reported by Adasch *et al.* (2006**a*,b*) and 1423 K, unreported reaction time, by Brutti *et al.* (2002).

With respect to the other published structures of the same rhombohedral family *viz*, MgB_17.4_ (Adasch *et al.*, 2006*b*) and MgB_20_ (Brutti *et al.*, 2002), Mg_2_B_25_ exhibits very small differences in the unit-cell parameters, but significant differences in the occupation of the interstitial magnesium sites, also with the identification of a new structural site never observed in the related metal borides. This site was labelled as Mg(N) by Giunchi *et al.* (2006). However, since the presence of partially occupied structural sites in the model obtained from XRPD data, some scepticism persisted in the literature about the true nature of the structure of Mg_2_B_25_. Here we present a re-examination of the crystal structure of Mg_2_B_25_ based on single-crystal X-ray diffraction data. The structural model previously reported by Giunchi *et al.* (2006) has been confirmed, although a much higher precision refinement was achieved, accompanied with the refinement of all atoms with anisotropic displacement parameters.

Mg_2_B_25_ adopts the *β*-boron structure and crystallizes isostructurally with other rhombohedral compounds of the boron-rich metal boride family (Fig. 1). Magnesium atoms occupy interstitial sites with partial occupancies that can be interpreted with the aid of the analysis of impossible interatomic Mg···Mg contacts.

### Experimental

A small crystal fragment was cut out from a polished thin section of a MgB_2_ bulk sample. This sample was obtained by the Mg-RLI process (Giunchi, 2003) under particular mild conditions (low temperature and short heat treatment) in order to have a consistent Mg_2_B_25_ fraction in the final sample. Thus, this small crystal does not have to be considered as a representative product of the Mg-RLI process, but just a sample synthesized *ad hoc*. The extracted crystal fragment was used for the X-ray single-crystal diffraction study.

### Refinement

The crystal structure refinement was performed starting from the atomic coordinates reported by Giunchi *et al.* (2006). Convergence was rapidly obtained for an anisotropic model of the structure. The site–occupancy factor of the Mg positions and of some of the B positions (*i.e.*, B4 and B13) was left free to vary.

### Crystal data

**Table:**

Mg_2_B_25_	*D*_x_ = 2.488 Mg m^−^^3^
*M**_r_* = 318.87	Mo *K*α radiation, λ = 0.71073 Å
Hexagonal, *R*3*m*	Cell parameters from 562 reflections
Hall symbol: -R 3 2"	θ = 10.6–26.2°
*a* = 11.0398 (5) Å	µ = 0.23 mm^−^^1^
*c* = 24.195 (1) Å	*T* = 298 K
*V* = 2553.75 (19) Å^3^	Block, black
*Z* = 12	0.08 × 0.07 × 0.07 mm
*F*(000) = 1788	

### Data collection

**Table:**

Oxford Xcalibur 3 CCD diffractometer	799 independent reflections
Radiation source: fine-focus sealed tube	724 reflections with *I* > 2σ(*I*)
Graphite monochromator	*R*_int_ = 0.045
ω scans	θ_max_ = 28.0°, θ_min_ = 2.3°
Absorption correction: gaussian (*ABSPACK*; Oxford Diffraction, 2006)	*h* = −14→14
*T*_min_ = 0.982, *T*_max_ = 0.984	*k* = −14→14
4794 measured reflections	*l* = −31→31

### Refinement

**Table:**

Refinement on *F*^2^	0 restraints
Least-squares matrix: full	Primary atom site location: structure-invariant direct methods
*R*[*F*^2^ > 2σ(*F*^2^)] = 0.021	Secondary atom site location: difference Fourier map
*wR*(*F*^2^) = 0.049	*w* = 1/[σ^2^(*F*_o_^2^) + (0.0342*P*)^2^] where *P* = (*F*_o_^2^ + 2*F*_c_^2^)/3
*S* = 1.13	(Δ/σ)_max_ = 0.001
799 reflections	Δρ_max_ = 0.14 e Å^−^^3^
122 parameters	Δρ_min_ = −0.31 e Å^−^^3^

### Special details

**Table:**

Geometry. All e.s.d.'s (except the e.s.d. in the dihedral angle between two l.s. planes) are estimated using the full covariance matrix. The cell e.s.d.'s are taken into account individually in the estimation of e.s.d.'s in distances, angles and torsion angles; correlations between e.s.d.'s in cell parameters are only used when they are defined by crystal symmetry. An approximate (isotropic) treatment of cell e.s.d.'s is used for estimating e.s.d.'s involving l.s. planes.
Refinement. Refinement of *F*^2^ against ALL reflections. The weighted *R*-factor *wR* and goodness of fit *S* are based on *F*^2^, conventional *R*-factors *R* are based on *F*, with *F* set to zero for negative *F*^2^. The threshold expression of *F*^2^ > σ(*F*^2^) is used only for calculating *R*-factors(gt) *etc*. and is not relevant to the choice of reflections for refinement. *R*-factors based on *F*^2^ are statistically about twice as large as those based on *F*, and *R*- factors based on ALL data will be even larger.

### Fractional atomic coordinates and isotropic or equivalent isotropic displacement parameters (Å^2^)

**Table:**

	*x*	*y*	*z*	*U*_iso_*/*U*_eq_	Occ. (<1)
B1	0.17051 (6)	0.16425 (6)	0.17121 (2)	0.01393 (14)	
B2	0.31095 (6)	0.29537 (6)	0.13087 (2)	0.01500 (14)	
B3	0.25656 (6)	0.21074 (6)	0.41962 (2)	0.01482 (14)	
B4	0.24302 (14)	0.2507 (2)	0.34557 (4)	0.0146 (4)	0.836 (7)
B5	0.05447 (4)	0.10893 (8)	0.94321 (3)	0.01410 (18)	
B6	0.08455 (4)	0.16910 (8)	0.01360 (3)	0.01402 (18)	
B7	0.10819 (4)	0.21638 (9)	0.88662 (3)	0.01461 (18)	
B8	0.17148 (4)	0.34295 (8)	0.02961 (3)	0.01510 (18)	
B9	0.13101 (4)	0.26201 (9)	0.76761 (3)	0.01487 (17)	
B10	0.10643 (4)	0.21286 (8)	0.70081 (3)	0.01434 (18)	
B11	0.05625 (4)	0.11249 (9)	0.32568 (3)	0.01443 (17)	
B12	0.08963 (4)	0.17926 (8)	0.40273 (3)	0.01459 (18)	
B13	0.05471 (9)	0.10941 (17)	0.55309 (6)	0.0136 (5)	0.493 (4)
B14	0.0000	0.0000	0.38677 (6)	0.0148 (3)	
B15	0.0000	0.0000	0.5000	0.0148 (4)	
MgN	0.12339 (3)	0.24679 (6)	0.25266 (2)	0.01846 (19)	0.5000 (14)
MgD	0.20089 (3)	0.40178 (6)	0.17677 (2)	0.0185 (2)	0.5019 (14)
MgE	0.0000	0.0000	0.23530 (4)	0.0190 (3)	0.501 (2)
MgF	0.37439 (15)	0.0000	0.0000	0.0182 (13)	0.166 (4)

### Atomic displacement parameters (Å^2^)

**Table:**

	*U*^11^	*U*^22^	*U*^33^	*U*^12^	*U*^13^	*U*^23^
B1	0.0141 (3)	0.0141 (3)	0.0135 (2)	0.0071 (2)	−0.00003 (19)	0.00011 (19)
B2	0.0150 (3)	0.0153 (3)	0.0149 (2)	0.0077 (2)	0.00008 (19)	0.00003 (18)
B3	0.0151 (3)	0.0146 (3)	0.0147 (3)	0.0074 (2)	0.0002 (2)	0.00001 (19)
B4	0.0147 (5)	0.0152 (9)	0.0140 (4)	0.0075 (6)	−0.0001 (3)	0.0001 (4)
B5	0.0141 (3)	0.0143 (4)	0.0140 (4)	0.00715 (19)	0.00002 (14)	0.0000 (3)
B6	0.0140 (3)	0.0147 (4)	0.0136 (4)	0.00735 (19)	0.00002 (12)	0.0000 (2)
B7	0.0149 (3)	0.0150 (4)	0.0140 (4)	0.0075 (2)	0.00010 (14)	0.0002 (3)
B8	0.0153 (3)	0.0150 (4)	0.0149 (4)	0.0075 (2)	−0.00003 (13)	−0.0001 (3)
B9	0.0151 (3)	0.0151 (4)	0.0144 (3)	0.00757 (19)	−0.00008 (13)	−0.0002 (3)
B10	0.0147 (3)	0.0146 (4)	0.0137 (4)	0.0073 (2)	0.00022 (14)	0.0004 (3)
B11	0.0144 (3)	0.0144 (4)	0.0144 (3)	0.00722 (18)	0.00000 (14)	0.0000 (3)
B12	0.0145 (3)	0.0147 (4)	0.0147 (4)	0.00735 (19)	0.00012 (14)	0.0002 (3)
B13	0.0138 (7)	0.0139 (8)	0.0133 (7)	0.0069 (4)	0.0004 (3)	0.0008 (5)
B14	0.0146 (4)	0.0146 (4)	0.0152 (6)	0.0073 (2)	0.000	0.000
B15	0.0149 (6)	0.0149 (6)	0.0146 (8)	0.0075 (3)	0.000	0.000
MgN	0.0187 (2)	0.0185 (3)	0.0181 (3)	0.00925 (15)	0.00013 (9)	0.00025 (19)
MgD	0.0185 (2)	0.0192 (3)	0.0180 (3)	0.00961 (16)	0.00006 (9)	0.00011 (18)
MgE	0.0190 (4)	0.0190 (4)	0.0188 (5)	0.00951 (19)	0.000	0.000
MgF	0.0187 (11)	0.017 (3)	0.0183 (9)	0.0086 (16)	0.0005 (7)	0.0010 (14)

### Geometric parameters (Å, º)

**Table:**

B1—B7^i^	1.7061 (9)	B11—B4^iii^	1.9145 (16)
B1—B1^ii^	1.7441 (12)	B11—B12	1.9703 (11)
B1—B2	1.7909 (8)	B11—MgN	2.1840 (9)
B1—B9^i^	1.9279 (10)	B11—MgE	2.4370 (12)
B1—B1^iii^	1.9515 (12)	B11—MgF^ix^	2.5328 (11)
B1—B2^iv^	2.0394 (8)	B11—MgF^xii^	2.5329 (11)
B1—MgN	2.3363 (7)	B12—B3^iii^	1.7446 (6)
B1—MgE	2.4130 (8)	B12—B14	1.7568 (8)
B1—MgD	2.4754 (8)	B12—B13^i^	1.8389 (11)
B2—B3^v^	1.7785 (8)	B12—B13^xiii^	1.8389 (11)
B2—B2^iv^	1.7842 (11)	B12—B4^iii^	2.0165 (16)
B2—B7^i^	1.8389 (8)	B12—MgD^x^	2.2623 (9)
B2—B9^vi^	1.9266 (8)	B12—MgN^x^	2.3166 (9)
B2—B1^iv^	2.0394 (8)	B12—MgF^ix^	2.6289 (11)
B2—MgD	2.3488 (7)	B12—MgF^xii^	2.6290 (11)
B2—MgD^vii^	2.3988 (6)	B13—B15	1.6566 (16)
B2—MgN^vii^	2.5803 (6)	B13—B14^xxv^	1.792 (2)
B3—B12	1.7446 (6)	B13—B3^xx^	1.8010 (15)
B3—B8^viii^	1.7667 (9)	B13—B3^xiii^	1.8010 (15)
B3—B2^viii^	1.7785 (8)	B13—B13^xv^	1.812 (3)
B3—B13^i^	1.8010 (15)	B13—B13^xvi^	1.812 (3)
B3—B3^ii^	1.8205 (12)	B13—B12^xiii^	1.8389 (11)
B3—B4	1.8690 (14)	B13—B12^i^	1.8389 (11)
B3—MgD^viii^	2.3723 (7)	B13—MgD^x^	2.1326 (17)
B3—MgF^ix^	2.4108 (6)	B13—MgD^viii^	2.4373 (9)
B3—MgD^x^	2.4382 (7)	B13—MgD^xxvii^	2.4374 (9)
B3—MgN^x^	2.6082 (7)	B14—B12^xvi^	1.7568 (8)
B4—MgF^ix^	0.848 (2)	B14—B12^xv^	1.7568 (8)
B4—B8^viii^	1.6753 (12)	B14—B13^i^	1.792 (2)
B4—B4^xi^	1.687 (4)	B14—B13^xiii^	1.792 (2)
B4—B10^i^	1.8378 (12)	B14—B13^xxv^	1.792 (2)
B4—B11	1.9145 (16)	B14—B11^xv^	1.8279 (15)
B4—B12	2.0165 (16)	B14—B11^xvi^	1.8279 (15)
B4—MgN	2.5964 (9)	B15—B13^xxv^	1.6566 (16)
B4—MgN^x^	2.6540 (10)	B15—B13^i^	1.6566 (16)
B4—MgF^xii^	2.8150 (16)	B15—B13^xiii^	1.6566 (16)
B5—B7	1.7117 (11)	B15—B13^xvi^	1.6566 (16)
B5—B6^i^	1.7626 (8)	B15—B13^xv^	1.6566 (16)
B5—B6^xiii^	1.7626 (8)	B15—MgD^xxviii^	2.5443 (6)
B5—B6^xiv^	1.7976 (11)	B15—MgD^viii^	2.5443 (6)
B5—B5^xv^	1.8039 (14)	B15—MgD^x^	2.5443 (6)
B5—B5^xvi^	1.8039 (14)	B15—MgD^xxix^	2.5443 (6)
B6—B8	1.7067 (12)	B15—MgD^xxx^	2.5444 (6)
B6—B6^xvii^	1.7455 (9)	B15—MgD^xxvii^	2.5444 (6)
B6—B6^xviii^	1.7455 (9)	MgN—B12^x^	2.3166 (9)
B6—B5^xiii^	1.7626 (8)	MgN—B1^iii^	2.3363 (7)
B6—B5^i^	1.7626 (8)	MgN—MgD	2.3596 (7)
B6—B5^xix^	1.7976 (11)	MgN—MgE	2.3966 (6)
B6—MgF^xv^	2.8691 (16)	MgN—B9^i^	2.4844 (6)
B6—MgF^xvii^	2.8691 (16)	MgN—B9^xiii^	2.4844 (6)
B7—B1^xiii^	1.7061 (9)	MgN—B10^i^	2.4848 (6)
B7—B1^xx^	1.7061 (9)	MgN—B10^xiii^	2.4848 (6)
B7—B9^xxi^	1.8250 (12)	MgN—MgF^ix^	2.5333 (10)
B7—B2^xx^	1.8389 (8)	MgN—MgF^xii^	2.5333 (10)
B7—B2^xiii^	1.8389 (8)	MgD—B13^x^	2.1326 (17)
B8—B4^xxii^	1.6753 (12)	MgD—B12^x^	2.2623 (9)
B8—B4^v^	1.6754 (12)	MgD—B2^iii^	2.3488 (7)
B8—B3^xxii^	1.7667 (9)	MgD—B3^xxii^	2.3723 (7)
B8—B3^v^	1.7668 (9)	MgD—B3^v^	2.3724 (7)
B8—B10^x^	1.8715 (11)	MgD—B2^iv^	2.3987 (6)
B8—MgF^xvii^	2.2078 (10)	MgD—B2^xxxi^	2.3988 (6)
B8—MgF^xv^	2.2078 (10)	MgD—B13^xxxii^	2.4373 (9)
B9—B10	1.6831 (11)	MgD—B13^v^	2.4373 (9)
B9—B7^xxi^	1.8250 (12)	MgD—B3^x^	2.4382 (7)
B9—B2^xxiii^	1.9266 (8)	MgE—MgN^xv^	2.3966 (6)
B9—B2^xxiv^	1.9267 (8)	MgE—MgN^xvi^	2.3966 (6)
B9—B1^xiii^	1.9279 (10)	MgE—B1^xxxiii^	2.4130 (8)
B9—B1^xx^	1.9279 (10)	MgE—B1^xvi^	2.4130 (8)
B9—MgN^xiii^	2.4844 (6)	MgE—B1^xv^	2.4130 (8)
B9—MgN^i^	2.4844 (6)	MgE—B1^ii^	2.4130 (8)
B9—MgE^xxv^	2.5060 (8)	MgE—B1^iii^	2.4130 (8)
B10—B4^xx^	1.8378 (12)	MgE—B11^xvi^	2.4370 (12)
B10—B4^xiii^	1.8378 (12)	MgE—B11^xv^	2.4370 (12)
B10—B8^x^	1.8715 (11)	MgF—B4^ix^	0.848 (2)
B10—B11^xiii^	1.8763 (7)	MgF—B4^xxxiv^	0.848 (2)
B10—B11^i^	1.8763 (7)	MgF—B8^xviii^	2.2078 (10)
B10—MgF^xxiv^	2.3515 (5)	MgF—B8^xvi^	2.2078 (10)
B10—MgF^xxvi^	2.3516 (5)	MgF—B10^xxx^	2.3516 (5)
B10—MgN^xiii^	2.4848 (6)	MgF—B10^vi^	2.3516 (5)
B10—MgN^i^	2.4848 (6)	MgF—B3^xxxiv^	2.4108 (6)
B10—MgE^xxv^	2.5556 (10)	MgF—B3^ix^	2.4108 (6)
B11—B14	1.8279 (15)	MgF—B11^ix^	2.5328 (11)
B11—B11^xv^	1.8629 (14)	MgF—B11^xxxv^	2.5329 (11)
B11—B11^xvi^	1.8629 (14)	MgF—MgN^ix^	2.5333 (10)
B11—B10^i^	1.8763 (7)	MgF—MgN^xxxv^	2.5333 (10)
B11—B10^xiii^	1.8763 (7)		
			
B7^i^—B1—B1^ii^	59.26 (2)	B11—B12—MgN^x^	115.67 (4)
B7^i^—B1—B2	63.40 (4)	B4—B12—MgN^x^	75.18 (5)
B1^ii^—B1—B2	112.05 (3)	B4^iii^—B12—MgN^x^	75.19 (5)
B7^i^—B1—B9^i^	106.37 (4)	MgD^x^—B12—MgN^x^	62.02 (3)
B1^ii^—B1—B9^i^	63.11 (2)	B3—B12—MgF^ix^	63.15 (4)
B2—B1—B9^i^	104.36 (4)	B3^iii^—B12—MgF^ix^	123.89 (5)
B7^i^—B1—B1^iii^	118.57 (3)	B14—B12—MgF^ix^	113.97 (5)
B1^ii^—B1—B1^iii^	120.0	B13^i^—B12—MgF^ix^	118.31 (6)
B2—B1—B1^iii^	117.49 (3)	B13^xiii^—B12—MgF^ix^	173.69 (6)
B9^i^—B1—B1^iii^	128.25 (3)	B11—B12—MgF^ix^	65.00 (3)
B7^i^—B1—B2^iv^	104.44 (4)	B4—B12—MgF^ix^	14.64 (6)
B1^ii^—B1—B2^iv^	109.25 (2)	B4^iii^—B12—MgF^ix^	73.26 (5)
B2—B1—B2^iv^	55.06 (3)	MgD^x^—B12—MgF^ix^	113.12 (3)
B9^i^—B1—B2^iv^	58.03 (3)	MgN^x^—B12—MgF^ix^	61.23 (2)
B1^iii^—B1—B2^iv^	125.95 (2)	B3—B12—MgF^xii^	123.89 (5)
B7^i^—B1—MgN	176.04 (4)	B3^iii^—B12—MgF^xii^	63.15 (4)
B1^ii^—B1—MgN	120.089 (18)	B14—B12—MgF^xii^	113.97 (5)
B2—B1—MgN	114.58 (3)	B13^i^—B12—MgF^xii^	173.69 (6)
B9^i^—B1—MgN	70.53 (3)	B13^xiii^—B12—MgF^xii^	118.31 (6)
B1^iii^—B1—MgN	65.314 (16)	B11—B12—MgF^xii^	65.00 (3)
B2^iv^—B1—MgN	71.90 (3)	B4—B12—MgF^xii^	73.26 (5)
B7^i^—B1—MgE	121.06 (4)	B4^iii^—B12—MgF^xii^	14.64 (6)
B1^ii^—B1—MgE	68.814 (15)	MgD^x^—B12—MgF^xii^	113.12 (3)
B2—B1—MgE	172.93 (4)	MgN^x^—B12—MgF^xii^	61.24 (2)
B9^i^—B1—MgE	69.50 (3)	MgF^ix^—B12—MgF^xii^	63.67 (6)
B1^iii^—B1—MgE	66.147 (15)	B15—B13—B14^xxv^	105.13 (9)
B2^iv^—B1—MgE	117.87 (4)	B15—B13—B3^xx^	141.03 (6)
MgN—B1—MgE	60.59 (2)	B14^xxv^—B13—B3^xx^	99.11 (7)
B7^i^—B1—MgD	121.42 (3)	B15—B13—B3^xiii^	141.03 (6)
B1^ii^—B1—MgD	172.571 (15)	B14^xxv^—B13—B3^xiii^	99.11 (7)
B2—B1—MgD	64.49 (3)	B3^xx^—B13—B3^xiii^	60.72 (7)
B9^i^—B1—MgD	110.71 (3)	B15—B13—B13^xv^	56.85 (4)
B1^iii^—B1—MgD	66.785 (15)	B14^xxv^—B13—B13^xv^	59.63 (5)
B2^iv^—B1—MgD	63.32 (2)	B3^xx^—B13—B13^xv^	158.31 (5)
MgN—B1—MgD	58.65 (2)	B3^xiii^—B13—B13^xv^	115.07 (4)
MgE—B1—MgD	113.75 (3)	B15—B13—B13^xvi^	56.85 (4)
B3^v^—B2—B2^iv^	128.25 (3)	B14^xxv^—B13—B13^xvi^	59.63 (5)
B3^v^—B2—B1	122.06 (4)	B3^xx^—B13—B13^xvi^	115.07 (4)
B2^iv^—B2—B1	69.56 (4)	B3^xiii^—B13—B13^xvi^	158.31 (5)
B3^v^—B2—B7^i^	117.24 (4)	B13^xv^—B13—B13^xvi^	60.0
B2^iv^—B2—B7^i^	109.96 (3)	B15—B13—B12^xiii^	112.69 (6)
B1—B2—B7^i^	56.05 (4)	B14^xxv^—B13—B12^xiii^	57.86 (5)
B3^v^—B2—B9^vi^	117.38 (4)	B3^xx^—B13—B12^xiii^	105.97 (8)
B2^iv^—B2—B9^vi^	104.67 (4)	B3^xiii^—B13—B12^xiii^	57.27 (4)
B1—B2—B9^vi^	104.63 (4)	B13^xv^—B13—B12^xiii^	60.49 (5)
B7^i^—B2—B9^vi^	57.92 (4)	B13^xvi^—B13—B12^xiii^	108.33 (5)
B3^v^—B2—B1^iv^	126.37 (4)	B15—B13—B12^i^	112.69 (6)
B2^iv^—B2—B1^iv^	55.37 (3)	B14^xxv^—B13—B12^i^	57.86 (5)
B1—B2—B1^iv^	108.97 (4)	B3^xx^—B13—B12^i^	57.27 (4)
B7^i^—B2—B1^iv^	103.61 (4)	B3^xiii^—B13—B12^i^	105.97 (8)
B9^vi^—B2—B1^iv^	58.09 (3)	B13^xv^—B13—B12^i^	108.33 (5)
B3^v^—B2—MgD	68.57 (3)	B13^xvi^—B13—B12^i^	60.49 (5)
B2^iv^—B2—MgD	69.42 (3)	B12^xiii^—B13—B12^i^	107.64 (9)
B1—B2—MgD	72.02 (3)	B15—B13—MgD^x^	83.34 (7)
B7^i^—B2—MgD	121.72 (4)	B14^xxv^—B13—MgD^x^	171.52 (10)
B9^vi^—B2—MgD	173.85 (4)	B3^xx^—B13—MgD^x^	73.64 (6)
B1^iv^—B2—MgD	117.63 (3)	B3^xiii^—B13—MgD^x^	73.64 (6)
B3^v^—B2—MgD^vii^	69.62 (3)	B13^xv^—B13—MgD^x^	127.13 (4)
B2^iv^—B2—MgD^vii^	66.45 (3)	B13^xvi^—B13—MgD^x^	127.13 (4)
B1—B2—MgD^vii^	126.89 (3)	B12^xiii^—B13—MgD^x^	119.24 (6)
B7^i^—B2—MgD^vii^	170.76 (4)	B12^i^—B13—MgD^x^	119.24 (6)
B9^vi^—B2—MgD^vii^	113.96 (4)	B15—B13—MgD^viii^	74.11 (4)
B1^iv^—B2—MgD^vii^	67.24 (3)	B14^xxv^—B13—MgD^viii^	113.53 (4)
MgD—B2—MgD^vii^	65.80 (3)	B3^xx^—B13—MgD^viii^	68.35 (3)
B3^v^—B2—MgN^vii^	70.80 (3)	B3^xiii^—B13—MgD^viii^	122.73 (7)
B2^iv^—B2—MgN^vii^	104.50 (4)	B13^xv^—B13—MgD^viii^	121.88 (4)
B1—B2—MgN^vii^	167.03 (4)	B13^xvi^—B13—MgD^viii^	68.18 (4)
B7^i^—B2—MgN^vii^	118.51 (4)	B12^xiii^—B13—MgD^viii^	169.64 (8)
B9^vi^—B2—MgN^vii^	65.03 (3)	B12^i^—B13—MgD^viii^	62.03 (3)
B1^iv^—B2—MgN^vii^	59.39 (2)	MgD^x^—B13—MgD^viii^	68.35 (4)
MgD—B2—MgN^vii^	117.48 (3)	B15—B13—MgD^xxvii^	74.11 (4)
MgD^vii^—B2—MgN^vii^	56.43 (2)	B14^xxv^—B13—MgD^xxvii^	113.53 (4)
B12—B3—B8^viii^	108.67 (5)	B3^xx^—B13—MgD^xxvii^	122.73 (7)
B12—B3—B2^viii^	122.77 (5)	B3^xiii^—B13—MgD^xxvii^	68.35 (3)
B8^viii^—B3—B2^viii^	120.34 (5)	B13^xv^—B13—MgD^xxvii^	68.18 (4)
B12—B3—B13^i^	62.46 (5)	B13^xvi^—B13—MgD^xxvii^	121.88 (4)
B8^viii^—B3—B13^i^	107.94 (5)	B12^xiii^—B13—MgD^xxvii^	62.03 (3)
B2^viii^—B3—B13^i^	120.86 (6)	B12^i^—B13—MgD^xxvii^	169.63 (8)
B12—B3—B3^ii^	109.21 (3)	MgD^x^—B13—MgD^xxvii^	68.35 (4)
B8^viii^—B3—B3^ii^	58.99 (2)	MgD^viii^—B13—MgD^xxvii^	128.28 (7)
B2^viii^—B3—B3^ii^	120.11 (3)	B12^xvi^—B14—B12	115.31 (4)
B13^i^—B3—B3^ii^	59.64 (3)	B12^xvi^—B14—B12^xv^	115.31 (4)
B12—B3—B4	67.74 (5)	B12—B14—B12^xv^	115.31 (4)
B8^viii^—B3—B4	54.79 (3)	B12^xvi^—B14—B13^i^	62.41 (4)
B2^viii^—B3—B4	119.05 (7)	B12—B14—B13^i^	62.41 (4)
B13^i^—B3—B4	115.90 (8)	B12^xv^—B14—B13^i^	113.02 (9)
B3^ii^—B3—B4	106.03 (5)	B12^xvi^—B14—B13^xiii^	113.02 (9)
B12—B3—MgD^viii^	111.91 (4)	B12—B14—B13^xiii^	62.41 (4)
B8^viii^—B3—MgD^viii^	120.43 (3)	B12^xv^—B14—B13^xiii^	62.41 (4)
B2^viii^—B3—MgD^viii^	67.17 (3)	B13^i^—B14—B13^xiii^	60.73 (9)
B13^i^—B3—MgD^viii^	59.60 (5)	B12^xvi^—B14—B13^xxv^	62.41 (4)
B3^ii^—B3—MgD^viii^	67.438 (14)	B12—B14—B13^xxv^	113.02 (9)
B4—B3—MgD^viii^	173.19 (6)	B12^xv^—B14—B13^xxv^	62.41 (4)
B12—B3—MgF^ix^	76.64 (5)	B13^i^—B14—B13^xxv^	60.73 (9)
B8^viii^—B3—MgF^ix^	61.54 (3)	B13^xiii^—B14—B13^xxv^	60.73 (9)
B2^viii^—B3—MgF^ix^	101.38 (3)	B12^xvi^—B14—B11^xv^	117.69 (5)
B13^i^—B3—MgF^ix^	132.10 (6)	B12—B14—B11^xv^	117.69 (5)
B3^ii^—B3—MgF^ix^	118.66 (2)	B12^xv^—B14—B11^xv^	66.65 (4)
B4—B3—MgF^ix^	17.68 (6)	B13^i^—B14—B11^xv^	179.67 (7)
MgD^viii^—B3—MgF^ix^	168.08 (4)	B13^xiii^—B14—B11^xv^	119.00 (5)
B12—B3—MgD^x^	62.94 (3)	B13^xxv^—B14—B11^xv^	119.00 (5)
B8^viii^—B3—MgD^x^	171.58 (4)	B12^xvi^—B14—B11^xvi^	66.65 (4)
B2^viii^—B3—MgD^x^	67.25 (3)	B12—B14—B11^xvi^	117.69 (5)
B13^i^—B3—MgD^x^	68.29 (4)	B12^xv^—B14—B11^xvi^	117.69 (5)
B3^ii^—B3—MgD^x^	121.747 (19)	B13^i^—B14—B11^xvi^	119.00 (5)
B4—B3—MgD^x^	119.30 (4)	B13^xiii^—B14—B11^xvi^	179.67 (8)
MgD^viii^—B3—MgD^x^	64.83 (2)	B13^xxv^—B14—B11^xvi^	119.00 (5)
MgF^ix^—B3—MgD^x^	114.86 (4)	B11^xv^—B14—B11^xvi^	61.27 (6)
B12—B3—MgN^x^	60.51 (3)	B12^xvi^—B14—B11	117.69 (5)
B8^viii^—B3—MgN^x^	121.91 (3)	B12—B14—B11	66.65 (4)
B2^viii^—B3—MgN^x^	69.11 (3)	B12^xv^—B14—B11	117.69 (5)
B13^i^—B3—MgN^x^	112.51 (4)	B13^i^—B14—B11	119.00 (5)
B3^ii^—B3—MgN^x^	169.708 (15)	B13^xiii^—B14—B11	119.00 (5)
B4—B3—MgN^x^	70.52 (4)	B13^xxv^—B14—B11	179.67 (8)
MgD^viii^—B3—MgN^x^	115.55 (2)	B11^xv^—B14—B11	61.27 (6)
MgF^ix^—B3—MgN^x^	60.47 (3)	B11^xvi^—B14—B11	61.27 (6)
MgD^x^—B3—MgN^x^	55.630 (19)	B13—B15—B13^xxv^	180.0
MgF^ix^—B4—B8^viii^	118.33 (13)	B13—B15—B13^i^	113.70 (9)
MgF^ix^—B4—B4^xi^	5.94 (12)	B13^xxv^—B15—B13^i^	66.30 (9)
B8^viii^—B4—B4^xi^	124.26 (7)	B13—B15—B13^xiii^	113.70 (9)
MgF^ix^—B4—B10^i^	117.38 (9)	B13^xxv^—B15—B13^xiii^	66.30 (9)
B8^viii^—B4—B10^i^	64.19 (5)	B13^i^—B15—B13^xiii^	66.30 (9)
B4^xi^—B4—B10^i^	119.25 (8)	B13—B15—B13^xvi^	66.30 (9)
MgF^ix^—B4—B3	120.31 (8)	B13^xxv^—B15—B13^xvi^	113.70 (9)
B8^viii^—B4—B3	59.50 (5)	B13^i^—B15—B13^xvi^	113.70 (9)
B4^xi^—B4—B3	121.87 (8)	B13^xiii^—B15—B13^xvi^	180.00 (7)
B10^i^—B4—B3	112.45 (10)	B13—B15—B13^xv^	66.30 (9)
MgF^ix^—B4—B11	128.71 (13)	B13^xxv^—B15—B13^xv^	113.70 (9)
B8^viii^—B4—B11	105.81 (11)	B13^i^—B15—B13^xv^	180.0
B4^xi^—B4—B11	123.93 (7)	B13^xiii^—B15—B13^xv^	113.70 (9)
B10^i^—B4—B11	59.96 (5)	B13^xvi^—B15—B13^xv^	66.30 (9)
B3—B4—B11	103.54 (10)	B13—B15—MgD^xxviii^	112.88 (2)
MgF^ix^—B4—B12	128.43 (11)	B13^xxv^—B15—MgD^xxviii^	67.12 (2)
B8^viii^—B4—B12	100.79 (10)	B13^i^—B15—MgD^xxviii^	123.64 (6)
B4^xi^—B4—B12	123.92 (6)	B13^xiii^—B15—MgD^xxviii^	67.12 (2)
B10^i^—B4—B12	109.08 (10)	B13^xvi^—B15—MgD^xxviii^	112.88 (2)
B3—B4—B12	53.19 (5)	B13^xv^—B15—MgD^xxviii^	56.36 (6)
B11—B4—B12	60.10 (6)	B13—B15—MgD^viii^	67.12 (2)
MgF^ix^—B4—MgN	76.30 (9)	B13^xxv^—B15—MgD^viii^	112.88 (2)
B8^viii^—B4—MgN	128.49 (5)	B13^i^—B15—MgD^viii^	56.36 (6)
B4^xi^—B4—MgN	73.12 (6)	B13^xiii^—B15—MgD^viii^	112.88 (2)
B10^i^—B4—MgN	65.58 (3)	B13^xvi^—B15—MgD^viii^	67.12 (2)
B3—B4—MgN	157.83 (9)	B13^xv^—B15—MgD^viii^	123.64 (6)
B11—B4—MgN	55.46 (3)	MgD^xxviii^—B15—MgD^viii^	180.00 (2)
B12—B4—MgN	105.46 (4)	B13—B15—MgD^x^	56.36 (6)
MgF^ix^—B4—MgN^x^	72.59 (9)	B13^xxv^—B15—MgD^x^	123.64 (6)
B8^viii^—B4—MgN^x^	123.78 (5)	B13^i^—B15—MgD^x^	67.12 (2)
B4^xi^—B4—MgN^x^	69.42 (6)	B13^xiii^—B15—MgD^x^	67.12 (2)
B10^i^—B4—MgN^x^	164.11 (8)	B13^xvi^—B15—MgD^x^	112.88 (2)
B3—B4—MgN^x^	67.89 (3)	B13^xv^—B15—MgD^x^	112.88 (2)
B11—B4—MgN^x^	104.20 (4)	MgD^xxviii^—B15—MgD^x^	119.089 (4)
B12—B4—MgN^x^	57.55 (3)	MgD^viii^—B15—MgD^x^	60.911 (4)
MgN—B4—MgN^x^	107.70 (4)	B13—B15—MgD^xxix^	123.64 (6)
MgF^ix^—B4—MgF^xii^	78.50 (14)	B13^xxv^—B15—MgD^xxix^	56.36 (6)
B8^viii^—B4—MgF^xii^	162.81 (12)	B13^i^—B15—MgD^xxix^	112.88 (2)
B4^xi^—B4—MgF^xii^	72.57 (5)	B13^xiii^—B15—MgD^xxix^	112.88 (2)
B10^i^—B4—MgF^xii^	112.77 (4)	B13^xvi^—B15—MgD^xxix^	67.12 (2)
B3—B4—MgF^xii^	110.31 (4)	B13^xv^—B15—MgD^xxix^	67.12 (2)
B11—B4—MgF^xii^	61.31 (3)	MgD^xxviii^—B15—MgD^xxix^	60.911 (4)
B12—B4—MgF^xii^	63.43 (3)	MgD^viii^—B15—MgD^xxix^	119.089 (4)
MgN—B4—MgF^xii^	55.650 (18)	MgD^x^—B15—MgD^xxix^	180.0
MgN^x^—B4—MgF^xii^	55.090 (17)	B13—B15—MgD^xxx^	112.88 (2)
B7—B5—B6^i^	123.62 (4)	B13^xxv^—B15—MgD^xxx^	67.12 (2)
B7—B5—B6^xiii^	123.62 (4)	B13^i^—B15—MgD^xxx^	67.12 (2)
B6^i^—B5—B6^xiii^	105.19 (6)	B13^xiii^—B15—MgD^xxx^	123.64 (6)
B7—B5—B6^xiv^	124.46 (6)	B13^xvi^—B15—MgD^xxx^	56.36 (6)
B6^i^—B5—B6^xiv^	58.71 (3)	B13^xv^—B15—MgD^xxx^	112.88 (2)
B6^xiii^—B5—B6^xiv^	58.71 (3)	MgD^xxviii^—B15—MgD^xxx^	119.087 (4)
B7—B5—B5^xv^	121.31 (3)	MgD^viii^—B15—MgD^xxx^	60.913 (4)
B6^i^—B5—B5^xv^	106.42 (3)	MgD^x^—B15—MgD^xxx^	119.087 (4)
B6^xiii^—B5—B5^xv^	59.22 (3)	MgD^xxix^—B15—MgD^xxx^	60.913 (4)
B6^xiv^—B5—B5^xv^	106.09 (3)	B13—B15—MgD^xxvii^	67.12 (2)
B7—B5—B5^xvi^	121.31 (3)	B13^xxv^—B15—MgD^xxvii^	112.88 (2)
B6^i^—B5—B5^xvi^	59.22 (3)	B13^i^—B15—MgD^xxvii^	112.88 (2)
B6^xiii^—B5—B5^xvi^	106.42 (3)	B13^xiii^—B15—MgD^xxvii^	56.36 (6)
B6^xiv^—B5—B5^xvi^	106.09 (3)	B13^xvi^—B15—MgD^xxvii^	123.64 (6)
B5^xv^—B5—B5^xvi^	60.0	B13^xv^—B15—MgD^xxvii^	67.12 (2)
B8—B6—B6^xvii^	122.451 (10)	MgD^xxviii^—B15—MgD^xxvii^	60.913 (4)
B8—B6—B6^xviii^	122.451 (10)	MgD^viii^—B15—MgD^xxvii^	119.087 (4)
B6^xvii^—B6—B6^xviii^	106.67 (5)	MgD^x^—B15—MgD^xxvii^	60.913 (4)
B8—B6—B5^xiii^	118.10 (5)	MgD^xxix^—B15—MgD^xxvii^	119.087 (4)
B6^xvii^—B6—B5^xiii^	61.65 (4)	MgD^xxx^—B15—MgD^xxvii^	180.0
B6^xviii^—B6—B5^xiii^	110.24 (4)	B11—MgN—B12^x^	119.24 (4)
B8—B6—B5^i^	118.10 (5)	B11—MgN—B1	118.93 (3)
B6^xvii^—B6—B5^i^	110.24 (4)	B12^x^—MgN—B1	115.77 (3)
B6^xviii^—B6—B5^i^	61.65 (4)	B11—MgN—B1^iii^	118.93 (3)
B5^xiii^—B6—B5^i^	61.56 (6)	B12^x^—MgN—B1^iii^	115.77 (3)
B8—B6—B5^xix^	121.78 (6)	B1—MgN—B1^iii^	49.37 (3)
B6^xvii^—B6—B5^xix^	59.64 (4)	B11—MgN—MgD	177.10 (4)
B6^xviii^—B6—B5^xix^	59.64 (4)	B12^x^—MgN—MgD	57.86 (3)
B5^xiii^—B6—B5^xix^	111.27 (5)	B1—MgN—MgD	63.62 (2)
B5^i^—B6—B5^xix^	111.27 (5)	B1^iii^—MgN—MgD	63.62 (2)
B8—B6—MgF^xv^	50.190 (19)	B11—MgN—MgE	64.09 (3)
B6^xvii^—B6—MgF^xv^	72.291 (16)	B12^x^—MgN—MgE	176.67 (4)
B6^xviii^—B6—MgF^xv^	148.36 (6)	B1—MgN—MgE	61.29 (3)
B5^xiii^—B6—MgF^xv^	97.17 (3)	B1^iii^—MgN—MgE	61.29 (3)
B5^i^—B6—MgF^xv^	149.54 (4)	MgD—MgN—MgE	118.81 (3)
B5^xix^—B6—MgF^xv^	96.33 (3)	B11—MgN—B9^i^	84.13 (2)
B8—B6—MgF^xvii^	50.190 (19)	B12^x^—MgN—B9^i^	117.75 (2)
B6^xvii^—B6—MgF^xvii^	148.36 (6)	B1—MgN—B9^i^	47.02 (2)
B6^xviii^—B6—MgF^xvii^	72.291 (16)	B1^iii^—MgN—B9^i^	92.73 (3)
B5^xiii^—B6—MgF^xvii^	149.54 (4)	MgD—MgN—B9^i^	97.25 (2)
B5^i^—B6—MgF^xvii^	97.17 (3)	MgE—MgN—B9^i^	61.754 (19)
B5^xix^—B6—MgF^xvii^	96.33 (3)	B11—MgN—B9^xiii^	84.13 (2)
MgF^xv^—B6—MgF^xvii^	92.16 (3)	B12^x^—MgN—B9^xiii^	117.76 (2)
B1^xiii^—B7—B1^xx^	61.48 (5)	B1—MgN—B9^xiii^	92.73 (3)
B1^xiii^—B7—B5	120.12 (5)	B1^iii^—MgN—B9^xiii^	47.02 (2)
B1^xx^—B7—B5	120.12 (5)	MgD—MgN—B9^xiii^	97.25 (2)
B1^xiii^—B7—B9^xxi^	112.88 (5)	MgE—MgN—B9^xiii^	61.75 (2)
B1^xx^—B7—B9^xxi^	112.88 (5)	B9^i^—MgN—B9^xiii^	121.67 (4)
B5—B7—B9^xxi^	117.39 (5)	B11—MgN—B10^i^	46.85 (2)
B1^xiii^—B7—B2^xx^	111.52 (4)	B12^x^—MgN—B10^i^	118.88 (2)
B1^xx^—B7—B2^xx^	60.55 (3)	B1—MgN—B10^i^	84.46 (2)
B5—B7—B2^xx^	118.63 (3)	B1^iii^—MgN—B10^i^	119.73 (3)
B9^xxi^—B7—B2^xx^	63.45 (3)	MgD—MgN—B10^i^	133.77 (2)
B1^xiii^—B7—B2^xiii^	60.55 (3)	MgE—MgN—B10^i^	63.11 (2)
B1^xx^—B7—B2^xiii^	111.52 (4)	B9^i^—MgN—B10^i^	39.60 (2)
B5—B7—B2^xiii^	118.63 (3)	B9^xiii^—MgN—B10^i^	117.92 (3)
B9^xxi^—B7—B2^xiii^	63.45 (3)	B11—MgN—B10^xiii^	46.85 (2)
B2^xx^—B7—B2^xiii^	114.24 (6)	B12^x^—MgN—B10^xiii^	118.88 (2)
B4^xxii^—B8—B4^v^	116.74 (11)	B1—MgN—B10^xiii^	119.73 (3)
B4^xxii^—B8—B6	113.05 (7)	B1^iii^—MgN—B10^xiii^	84.46 (2)
B4^v^—B8—B6	113.05 (7)	MgD—MgN—B10^xiii^	133.77 (2)
B4^xxii^—B8—B3^xxii^	65.71 (4)	MgE—MgN—B10^xiii^	63.11 (2)
B4^v^—B8—B3^xxii^	117.78 (7)	B9^i^—MgN—B10^xiii^	117.92 (3)
B6—B8—B3^xxii^	122.01 (5)	B9^xiii^—MgN—B10^xiii^	39.60 (2)
B4^xxii^—B8—B3^v^	117.78 (7)	B10^i^—MgN—B10^xiii^	90.36 (4)
B4^v^—B8—B3^v^	65.71 (4)	B11—MgN—MgF^ix^	64.45 (2)
B6—B8—B3^v^	122.01 (5)	B12^x^—MgN—MgF^ix^	65.47 (2)
B3^xxii^—B8—B3^v^	62.03 (4)	B1—MgN—MgF^ix^	122.12 (3)
B4^xxii^—B8—B10^x^	62.12 (4)	B1^iii^—MgN—MgF^ix^	171.44 (4)
B4^v^—B8—B10^x^	62.12 (4)	MgD—MgN—MgF^ix^	113.23 (2)
B6—B8—B10^x^	111.38 (5)	MgE—MgN—MgF^ix^	117.17 (3)
B3^xxii^—B8—B10^x^	115.72 (5)	B9^i^—MgN—MgF^ix^	79.61 (3)
B3^v^—B8—B10^x^	115.72 (5)	B9^xiii^—MgN—MgF^ix^	140.86 (4)
B4^xxii^—B8—MgF^xvii^	130.76 (6)	B10^i^—MgN—MgF^ix^	55.88 (2)
B4^v^—B8—MgF^xvii^	19.76 (7)	B10^xiii^—MgN—MgF^ix^	102.45 (3)
B6—B8—MgF^xvii^	93.38 (4)	B11—MgN—MgF^xii^	64.45 (2)
B3^xxii^—B8—MgF^xvii^	133.21 (5)	B12^x^—MgN—MgF^xii^	65.47 (2)
B3^v^—B8—MgF^xvii^	73.74 (3)	B1—MgN—MgF^xii^	171.44 (4)
B10^x^—B8—MgF^xvii^	69.85 (2)	B1^iii^—MgN—MgF^xii^	122.11 (3)
B4^xxii^—B8—MgF^xv^	19.76 (7)	MgD—MgN—MgF^xii^	113.23 (2)
B4^v^—B8—MgF^xv^	130.76 (6)	MgE—MgN—MgF^xii^	117.17 (3)
B6—B8—MgF^xv^	93.38 (4)	B9^i^—MgN—MgF^xii^	140.86 (4)
B3^xxii^—B8—MgF^xv^	73.75 (3)	B9^xiii^—MgN—MgF^xii^	79.61 (3)
B3^v^—B8—MgF^xv^	133.21 (5)	B10^i^—MgN—MgF^xii^	102.45 (3)
B10^x^—B8—MgF^xv^	69.85 (2)	B10^xiii^—MgN—MgF^xii^	55.88 (3)
MgF^xvii^—B8—MgF^xv^	138.80 (4)	MgF^ix^—MgN—MgF^xii^	66.38 (6)
B10—B9—B7^xxi^	115.70 (6)	B13^x^—MgD—B12^x^	114.60 (5)
B10—B9—B2^xxiii^	119.39 (3)	B13^x^—MgD—B2^iii^	87.88 (3)
B7^xxi^—B9—B2^xxiii^	58.63 (3)	B12^x^—MgD—B2^iii^	126.690 (19)
B10—B9—B2^xxiv^	119.39 (3)	B13^x^—MgD—B2	87.87 (3)
B7^xxi^—B9—B2^xxiv^	58.63 (3)	B12^x^—MgD—B2	126.688 (19)
B2^xxiii^—B9—B2^xxiv^	106.56 (5)	B2^iii^—MgD—B2	100.24 (3)
B10—B9—B1^xiii^	127.63 (5)	B13^x^—MgD—MgN	174.71 (5)
B7^xxi^—B9—B1^xiii^	108.72 (5)	B12^x^—MgD—MgN	60.12 (3)
B2^xxiii^—B9—B1^xiii^	63.89 (3)	B2^iii^—MgD—MgN	95.50 (2)
B2^xxiv^—B9—B1^xiii^	106.56 (4)	B2—MgD—MgN	95.50 (2)
B10—B9—B1^xx^	127.63 (5)	B13^x^—MgD—B3^xxii^	46.76 (4)
B7^xxi^—B9—B1^xx^	108.72 (5)	B12^x^—MgD—B3^xxii^	148.02 (3)
B2^xxiii^—B9—B1^xx^	106.56 (4)	B2^iii^—MgD—B3^xxii^	44.26 (2)
B2^xxiv^—B9—B1^xx^	63.89 (3)	B2—MgD—B3^xxii^	82.68 (2)
B1^xiii^—B9—B1^xx^	53.79 (4)	MgN—MgD—B3^xxii^	137.67 (3)
B10—B9—MgN^xiii^	70.21 (3)	B13^x^—MgD—B3^v^	46.76 (4)
B7^xxi^—B9—MgN^xiii^	123.97 (2)	B12^x^—MgD—B3^v^	148.01 (3)
B2^xxiii^—B9—MgN^xiii^	70.30 (2)	B2^iii^—MgD—B3^v^	82.69 (2)
B2^xxiv^—B9—MgN^xiii^	168.97 (5)	B2—MgD—B3^v^	44.26 (2)
B1^xiii^—B9—MgN^xiii^	62.45 (3)	MgN—MgD—B3^v^	137.67 (3)
B1^xx^—B9—MgN^xiii^	106.35 (4)	B3^xxii^—MgD—B3^v^	45.13 (3)
B10—B9—MgN^i^	70.21 (3)	B13^x^—MgD—B2^iv^	114.786 (18)
B7^xxi^—B9—MgN^i^	123.96 (2)	B12^x^—MgD—B2^iv^	83.08 (2)
B2^xxiii^—B9—MgN^i^	168.97 (5)	B2^iii^—MgD—B2^iv^	132.27 (3)
B2^xxiv^—B9—MgN^i^	70.30 (2)	B2—MgD—B2^iv^	44.13 (3)
B1^xiii^—B9—MgN^i^	106.35 (4)	MgN—MgD—B2^iv^	65.670 (17)
B1^xx^—B9—MgN^i^	62.45 (2)	B3^xxii^—MgD—B2^iv^	126.73 (2)
MgN^xiii^—B9—MgN^i^	110.67 (4)	B3^v^—MgD—B2^iv^	84.42 (2)
B10—B9—MgE^xxv^	72.18 (5)	B13^x^—MgD—B2^xxxi^	114.788 (17)
B7^xxi^—B9—MgE^xxv^	172.12 (5)	B12^x^—MgD—B2^xxxi^	83.09 (2)
B2^xxiii^—B9—MgE^xxv^	118.38 (3)	B2^iii^—MgD—B2^xxxi^	44.13 (3)
B2^xxiv^—B9—MgE^xxv^	118.38 (3)	B2—MgD—B2^xxxi^	132.27 (3)
B1^xiii^—B9—MgE^xxv^	64.40 (4)	MgN—MgD—B2^xxxi^	65.671 (17)
B1^xx^—B9—MgE^xxv^	64.40 (4)	B3^xxii^—MgD—B2^xxxi^	84.42 (2)
MgN^xiii^—B9—MgE^xxv^	57.40 (2)	B3^v^—MgD—B2^xxxi^	126.73 (2)
MgN^i^—B9—MgE^xxv^	57.40 (2)	B2^iv^—MgD—B2^xxxi^	129.90 (4)
B9—B10—B4^xx^	122.86 (7)	B13^x^—MgD—B13^xxxii^	74.41 (7)
B9—B10—B4^xiii^	122.86 (7)	B12^x^—MgD—B13^xxxii^	45.88 (4)
B4^xx^—B10—B4^xiii^	101.83 (10)	B2^iii^—MgD—B13^xxxii^	105.88 (4)
B9—B10—B8^x^	129.29 (6)	B2—MgD—B13^xxxii^	147.57 (5)
B4^xx^—B10—B8^x^	53.69 (3)	MgN—MgD—B13^xxxii^	100.70 (4)
B4^xiii^—B10—B8^x^	53.69 (3)	B3^xxii^—MgD—B13^xxxii^	102.92 (4)
B9—B10—B11^xiii^	123.43 (5)	B3^v^—MgD—B13^xxxii^	120.58 (4)
B4^xx^—B10—B11^xiii^	107.57 (7)	B2^iv^—MgD—B13^xxxii^	120.15 (4)
B4^xiii^—B10—B11^xiii^	62.05 (5)	B2^xxxi^—MgD—B13^xxxii^	80.14 (4)
B8^x^—B10—B11^xiii^	99.81 (4)	B13^x^—MgD—B13^v^	74.41 (7)
B9—B10—B11^i^	123.43 (5)	B12^x^—MgD—B13^v^	45.88 (4)
B4^xx^—B10—B11^i^	62.05 (5)	B2^iii^—MgD—B13^v^	147.58 (5)
B4^xiii^—B10—B11^i^	107.57 (7)	B2—MgD—B13^v^	105.88 (4)
B8^x^—B10—B11^i^	99.81 (4)	MgN—MgD—B13^v^	100.70 (4)
B11^xiii^—B10—B11^i^	59.53 (6)	B3^xxii^—MgD—B13^v^	120.58 (4)
B9—B10—MgF^xxiv^	104.31 (3)	B3^v^—MgD—B13^v^	102.92 (4)
B4^xx^—B10—MgF^xxiv^	114.64 (5)	B2^iv^—MgD—B13^v^	80.14 (4)
B4^xiii^—B10—MgF^xxiv^	18.67 (6)	B2^xxxi^—MgD—B13^v^	120.15 (4)
B8^x^—B10—MgF^xxiv^	61.81 (3)	B13^xxxii^—MgD—B13^v^	43.64 (8)
B11^xiii^—B10—MgF^xxiv^	72.62 (5)	B13^x^—MgD—B3^x^	110.61 (4)
B11^i^—B10—MgF^xxiv^	125.02 (5)	B12^x^—MgD—B3^x^	43.372 (16)
B9—B10—MgF^xxvi^	104.31 (3)	B2^iii^—MgD—B3^x^	84.05 (2)
B4^xx^—B10—MgF^xxvi^	18.67 (6)	B2—MgD—B3^x^	161.26 (3)
B4^xiii^—B10—MgF^xxvi^	114.64 (5)	MgN—MgD—B3^x^	65.84 (2)
B8^x^—B10—MgF^xxvi^	61.81 (3)	B3^xxii^—MgD—B3^x^	111.948 (17)
B11^xiii^—B10—MgF^xxvi^	125.02 (5)	B3^v^—MgD—B3^x^	154.04 (3)
B11^i^—B10—MgF^xxvi^	72.62 (5)	B2^iv^—MgD—B3^x^	120.70 (3)
MgF^xxiv^—B10—MgF^xxvi^	123.00 (7)	B2^xxxi^—MgD—B3^x^	43.13 (2)
B9—B10—MgN^xiii^	70.19 (3)	B13^xxxii^—MgD—B3^x^	43.36 (4)
B4^xx^—B10—MgN^xiii^	165.69 (7)	B13^v^—MgD—B3^x^	77.40 (4)
B4^xiii^—B10—MgN^xiii^	72.08 (4)	MgN—MgE—MgN^xv^	116.996 (14)
B8^x^—B10—MgN^xiii^	124.636 (18)	MgN—MgE—MgN^xvi^	116.996 (14)
B11^xiii^—B10—MgN^xiii^	58.12 (3)	MgN^xv^—MgE—MgN^xvi^	116.996 (14)
B11^i^—B10—MgN^xiii^	106.76 (4)	MgN—MgE—B1^xxxiii^	141.06 (3)
MgF^xxiv^—B10—MgN^xiii^	63.10 (3)	MgN^xv^—MgE—B1^xxxiii^	58.125 (18)
MgF^xxvi^—B10—MgN^xiii^	173.22 (4)	MgN^xvi^—MgE—B1^xxxiii^	95.06 (2)
B9—B10—MgN^i^	70.19 (3)	MgN—MgE—B1^xvi^	141.06 (3)
B4^xx^—B10—MgN^i^	72.08 (4)	MgN^xv^—MgE—B1^xvi^	95.06 (2)
B4^xiii^—B10—MgN^i^	165.69 (7)	MgN^xvi^—MgE—B1^xvi^	58.125 (18)
B8^x^—B10—MgN^i^	124.633 (19)	B1^xxxiii^—MgE—B1^xvi^	42.37 (3)
B11^xiii^—B10—MgN^i^	106.76 (4)	MgN—MgE—B1^xv^	95.06 (2)
B11^i^—B10—MgN^i^	58.12 (3)	MgN^xv^—MgE—B1^xv^	58.125 (18)
MgF^xxiv^—B10—MgN^i^	173.22 (3)	MgN^xvi^—MgE—B1^xv^	141.06 (3)
MgF^xxvi^—B10—MgN^i^	63.10 (3)	B1^xxxiii^—MgE—B1^xv^	47.71 (3)
MgN^xiii^—B10—MgN^i^	110.64 (4)	B1^xvi^—MgE—B1^xv^	83.14 (3)
B9—B10—MgE^xxv^	68.99 (4)	MgN—MgE—B1	58.125 (18)
B4^xx^—B10—MgE^xxv^	119.62 (3)	MgN^xv^—MgE—B1	141.06 (3)
B4^xiii^—B10—MgE^xxv^	119.62 (3)	MgN^xvi^—MgE—B1	95.06 (2)
B8^x^—B10—MgE^xxv^	161.71 (5)	B1^xxxiii^—MgE—B1	99.95 (4)
B11^xiii^—B10—MgE^xxv^	64.60 (3)	B1^xvi^—MgE—B1	83.14 (3)
B11^i^—B10—MgE^xxv^	64.60 (4)	B1^xv^—MgE—B1	83.14 (3)
MgF^xxiv^—B10—MgE^xxv^	118.01 (4)	MgN—MgE—B1^ii^	95.06 (2)
MgF^xxvi^—B10—MgE^xxv^	118.01 (4)	MgN^xv^—MgE—B1^ii^	141.06 (3)
MgN^xiii^—B10—MgE^xxv^	56.760 (19)	MgN^xvi^—MgE—B1^ii^	58.125 (18)
MgN^i^—B10—MgE^xxv^	56.760 (19)	B1^xxxiii^—MgE—B1^ii^	83.14 (3)
B14—B11—B11^xv^	59.37 (3)	B1^xvi^—MgE—B1^ii^	47.71 (3)
B14—B11—B11^xvi^	59.37 (3)	B1^xv^—MgE—B1^ii^	99.95 (4)
B11^xv^—B11—B11^xvi^	60.0	B1—MgE—B1^ii^	42.37 (3)
B14—B11—B10^i^	104.96 (3)	MgN—MgE—B1^iii^	58.125 (18)
B11^xv^—B11—B10^i^	116.29 (3)	MgN^xv^—MgE—B1^iii^	95.06 (2)
B11^xvi^—B11—B10^i^	60.24 (3)	MgN^xvi^—MgE—B1^iii^	141.06 (3)
B14—B11—B10^xiii^	104.96 (3)	B1^xxxiii^—MgE—B1^iii^	83.14 (3)
B11^xv^—B11—B10^xiii^	60.24 (3)	B1^xvi^—MgE—B1^iii^	99.95 (4)
B11^xvi^—B11—B10^xiii^	116.29 (3)	B1^xv^—MgE—B1^iii^	42.37 (3)
B10^i^—B11—B10^xiii^	139.88 (6)	B1—MgE—B1^iii^	47.71 (3)
B14—B11—B4	101.71 (6)	B1^ii^—MgE—B1^iii^	83.14 (3)
B11^xv^—B11—B4	159.56 (6)	MgN—MgE—B11	53.72 (3)
B11^xvi^—B11—B4	104.99 (5)	MgN^xv^—MgE—B11	93.44 (3)
B10^i^—B11—B4	57.99 (3)	MgN^xvi^—MgE—B11	93.44 (3)
B10^xiii^—B11—B4	138.11 (7)	B1^xxxiii^—MgE—B11	151.02 (3)
B14—B11—B4^iii^	101.71 (6)	B1^xvi^—MgE—B11	151.02 (3)
B11^xv^—B11—B4^iii^	104.99 (5)	B1^xv^—MgE—B11	124.451 (19)
B11^xvi^—B11—B4^iii^	159.56 (6)	B1—MgE—B11	106.82 (2)
B10^i^—B11—B4^iii^	138.11 (7)	B1^ii^—MgE—B11	124.451 (19)
B10^xiii^—B11—B4^iii^	57.99 (3)	B1^iii^—MgE—B11	106.82 (2)
B4—B11—B4^iii^	85.45 (7)	MgN—MgE—B11^xvi^	93.44 (3)
B14—B11—B12	54.95 (4)	MgN^xv^—MgE—B11^xvi^	93.44 (3)
B11^xv^—B11—B12	106.29 (3)	MgN^xvi^—MgE—B11^xvi^	53.72 (3)
B11^xvi^—B11—B12	106.29 (3)	B1^xxxiii^—MgE—B11^xvi^	124.451 (19)
B10^i^—B11—B12	109.46 (3)	B1^xvi^—MgE—B11^xvi^	106.82 (2)
B10^xiii^—B11—B12	109.46 (3)	B1^xv^—MgE—B11^xvi^	151.02 (3)
B4—B11—B12	62.52 (4)	B1—MgE—B11^xvi^	124.451 (19)
B4^iii^—B11—B12	62.52 (4)	B1^ii^—MgE—B11^xvi^	106.82 (2)
B14—B11—MgN	179.96 (4)	B1^iii^—MgE—B11^xvi^	151.02 (3)
B11^xv^—B11—MgN	120.61 (2)	B11—MgE—B11^xvi^	44.94 (4)
B11^xvi^—B11—MgN	120.61 (2)	MgN—MgE—B11^xv^	93.44 (3)
B10^i^—B11—MgN	75.04 (3)	MgN^xv^—MgE—B11^xv^	53.72 (3)
B10^xiii^—B11—MgN	75.04 (3)	MgN^xvi^—MgE—B11^xv^	93.44 (3)
B4—B11—MgN	78.32 (6)	B1^xxxiii^—MgE—B11^xv^	106.82 (2)
B4^iii^—B11—MgN	78.32 (6)	B1^xvi^—MgE—B11^xv^	124.451 (19)
B12—B11—MgN	125.09 (5)	B1^xv^—MgE—B11^xv^	106.82 (2)
B14—B11—MgE	117.77 (5)	B1—MgE—B11^xv^	151.02 (3)
B11^xv^—B11—MgE	67.529 (18)	B1^ii^—MgE—B11^xv^	151.02 (3)
B11^xvi^—B11—MgE	67.529 (18)	B1^iii^—MgE—B11^xv^	124.451 (19)
B10^i^—B11—MgE	71.32 (3)	B11—MgE—B11^xv^	44.94 (4)
B10^xiii^—B11—MgE	71.32 (3)	B11^xvi^—MgE—B11^xv^	44.94 (4)
B4—B11—MgE	122.02 (4)	B4^ix^—MgF—B4^xxxiv^	168.1 (2)
B4^iii^—B11—MgE	122.02 (4)	B4^ix^—MgF—B8^xviii^	41.91 (9)
B12—B11—MgE	172.71 (5)	B4^xxxiv^—MgF—B8^xviii^	149.94 (15)
MgN—B11—MgE	62.20 (3)	B4^ix^—MgF—B8^xvi^	149.94 (15)
B14—B11—MgF^ix^	115.56 (4)	B4^xxxiv^—MgF—B8^xvi^	41.91 (9)
B11^xv^—B11—MgF^ix^	174.67 (2)	B8^xviii^—MgF—B8^xvi^	108.26 (8)
B11^xvi^—B11—MgF^ix^	116.63 (3)	B4^ix^—MgF—B10^xxx^	138.86 (5)
B10^i^—B11—MgF^ix^	62.39 (4)	B4^xxxiv^—MgF—B10^xxx^	43.94 (5)
B10^xiii^—B11—MgF^ix^	124.40 (5)	B8^xviii^—MgF—B10^xxx^	118.52 (6)
B4—B11—MgF^ix^	15.14 (6)	B8^xvi^—MgF—B10^xxx^	48.34 (3)
B4^iii^—B11—MgF^ix^	77.15 (5)	B4^ix^—MgF—B10^vi^	43.94 (5)
B12—B11—MgF^ix^	70.17 (3)	B4^xxxiv^—MgF—B10^vi^	138.86 (5)
MgN—B11—MgF^ix^	64.47 (3)	B8^xviii^—MgF—B10^vi^	48.34 (3)
MgE—B11—MgF^ix^	115.69 (3)	B8^xvi^—MgF—B10^vi^	118.52 (6)
B14—B11—MgF^xii^	115.56 (4)	B10^xxx^—MgF—B10^vi^	161.61 (9)
B11^xv^—B11—MgF^xii^	116.63 (3)	B4^ix^—MgF—B3^xxxiv^	140.10 (5)
B11^xvi^—B11—MgF^xii^	174.67 (2)	B4^xxxiv^—MgF—B3^xxxiv^	42.02 (5)
B10^i^—B11—MgF^xii^	124.40 (5)	B8^xviii^—MgF—B3^xxxiv^	124.98 (6)
B10^xiii^—B11—MgF^xii^	62.38 (4)	B8^xvi^—MgF—B3^xxxiv^	44.71 (3)
B4—B11—MgF^xii^	77.15 (5)	B10^xxx^—MgF—B3^xxxiv^	80.62 (2)
B4^iii^—B11—MgF^xii^	15.14 (6)	B10^vi^—MgF—B3^xxxiv^	97.21 (3)
B12—B11—MgF^xii^	70.17 (3)	B4^ix^—MgF—B3^ix^	42.02 (5)
MgN—B11—MgF^xii^	64.47 (3)	B4^xxxiv^—MgF—B3^ix^	140.10 (5)
MgE—B11—MgF^xii^	115.69 (3)	B8^xviii^—MgF—B3^ix^	44.71 (3)
MgF^ix^—B11—MgF^xii^	66.39 (7)	B8^xvi^—MgF—B3^ix^	124.98 (6)
B3—B12—B3^iii^	146.17 (6)	B10^xxx^—MgF—B3^ix^	97.21 (3)
B3—B12—B14	102.69 (3)	B10^vi^—MgF—B3^ix^	80.62 (2)
B3^iii^—B12—B14	102.69 (3)	B3^xxxiv^—MgF—B3^ix^	166.54 (8)
B3—B12—B13^i^	60.28 (6)	B4^ix^—MgF—B11^ix^	36.14 (8)
B3^iii^—B12—B13^i^	116.54 (6)	B4^xxxiv^—MgF—B11^ix^	133.97 (13)
B14—B12—B13^i^	59.73 (6)	B8^xviii^—MgF—B11^ix^	74.07 (2)
B3—B12—B13^xiii^	116.54 (6)	B8^xvi^—MgF—B11^ix^	156.36 (3)
B3^iii^—B12—B13^xiii^	60.28 (6)	B10^xxx^—MgF—B11^ix^	151.91 (6)
B14—B12—B13^xiii^	59.73 (6)	B10^vi^—MgF—B11^ix^	44.99 (2)
B13^i^—B12—B13^xiii^	59.03 (10)	B3^xxxiv^—MgF—B11^ix^	113.96 (3)
B3—B12—B11	106.11 (3)	B3^ix^—MgF—B11^ix^	73.87 (3)
B3^iii^—B12—B11	106.11 (3)	B4^ix^—MgF—B11^xxxv^	133.97 (13)
B14—B12—B11	58.40 (6)	B4^xxxiv^—MgF—B11^xxxv^	36.14 (8)
B13^i^—B12—B11	109.88 (5)	B8^xviii^—MgF—B11^xxxv^	156.36 (3)
B13^xiii^—B12—B11	109.88 (5)	B8^xvi^—MgF—B11^xxxv^	74.07 (2)
B3—B12—B4	59.07 (3)	B10^xxx^—MgF—B11^xxxv^	44.99 (2)
B3^iii^—B12—B4	135.93 (6)	B10^vi^—MgF—B11^xxxv^	151.91 (6)
B14—B12—B4	100.36 (6)	B3^xxxiv^—MgF—B11^xxxv^	73.87 (3)
B13^i^—B12—B4	107.50 (6)	B3^ix^—MgF—B11^xxxv^	113.96 (3)
B13^xiii^—B12—B4	159.29 (8)	B11^ix^—MgF—B11^xxxv^	113.61 (7)
B11—B12—B4	57.38 (4)	B4^ix^—MgF—MgN^ix^	84.73 (8)
B3—B12—B4^iii^	135.93 (6)	B4^xxxiv^—MgF—MgN^ix^	88.78 (8)
B3^iii^—B12—B4^iii^	59.07 (3)	B8^xviii^—MgF—MgN^ix^	109.16 (2)
B14—B12—B4^iii^	100.36 (6)	B8^xvi^—MgF—MgN^ix^	108.26 (2)
B13^i^—B12—B4^iii^	159.29 (8)	B10^xxx^—MgF—MgN^ix^	131.26 (4)
B13^xiii^—B12—B4^iii^	107.50 (6)	B10^vi^—MgF—MgN^ix^	61.02 (2)
B11—B12—B4^iii^	57.38 (4)	B3^xxxiv^—MgF—MgN^ix^	63.62 (2)
B4—B12—B4^iii^	80.20 (6)	B3^ix^—MgF—MgN^ix^	124.93 (4)
B3—B12—MgD^x^	73.69 (3)	B11^ix^—MgF—MgN^ix^	51.08 (3)
B3^iii^—B12—MgD^x^	73.69 (3)	B11^xxxv^—MgF—MgN^ix^	91.66 (4)
B14—B12—MgD^x^	123.91 (6)	B4^ix^—MgF—MgN^xxxv^	88.78 (8)
B13^i^—B12—MgD^x^	72.09 (5)	B4^xxxiv^—MgF—MgN^xxxv^	84.72 (8)
B13^xiii^—B12—MgD^x^	72.09 (5)	B8^xviii^—MgF—MgN^xxxv^	108.26 (2)
B11—B12—MgD^x^	177.69 (5)	B8^xvi^—MgF—MgN^xxxv^	109.16 (2)
B4—B12—MgD^x^	121.11 (4)	B10^xxx^—MgF—MgN^xxxv^	61.02 (2)
B4^iii^—B12—MgD^x^	121.11 (4)	B10^vi^—MgF—MgN^xxxv^	131.26 (4)
B3—B12—MgN^x^	78.53 (3)	B3^xxxiv^—MgF—MgN^xxxv^	124.93 (4)
B3^iii^—B12—MgN^x^	78.53 (3)	B3^ix^—MgF—MgN^xxxv^	63.62 (2)
B14—B12—MgN^x^	174.07 (6)	B11^ix^—MgF—MgN^xxxv^	91.66 (4)
B13^i^—B12—MgN^x^	125.07 (6)	B11^xxxv^—MgF—MgN^xxxv^	51.07 (3)
B13^xiii^—B12—MgN^x^	125.08 (6)	MgN^ix^—MgF—MgN^xxxv^	113.62 (6)

Symmetry codes: (i) *y*, −*x*+*y*, −*z*+1; (ii) *x*, *x*−*y*, *z*; (iii) −*x*+*y*, *y*, *z*; (iv) −*x*+2/3, −*x*+*y*+1/3, −*z*+1/3; (v) −*x*+*y*+1/3, −*x*+2/3, *z*−1/3; (vi) −*y*+2/3, *x*−*y*+1/3, *z*−2/3; (vii) *x*−*y*+2/3, *x*+1/3, −*z*+1/3; (viii) −*y*+2/3, *x*−*y*+1/3, *z*+1/3; (ix) −*x*+2/3, −*y*+1/3, −*z*+1/3; (x) −*x*+1/3, −*y*+2/3, −*z*+2/3; (xi) *x*−*y*+1/3, −*y*+2/3, −*z*+2/3; (xii) *x*−1/3, *y*+1/3, *z*+1/3; (xiii) *x*−*y*, *x*, −*z*+1; (xiv) *x*, *y*, *z*+1; (xv) −*y*, *x*−*y*, *z*; (xvi) −*x*+*y*, −*x*, *z*; (xvii) *x*−*y*, *x*, −*z*; (xviii) *y*, −*x*+*y*, −*z*; (xix) *x*, *y*, *z*−1; (xx) *y*, *x*, −*z*+1; (xxi) −*x*+1/3, −*y*+2/3, −*z*+5/3; (xxii) −*y*+1/3, −*x*+2/3, *z*−1/3; (xxiii) −*y*+1/3, −*x*+2/3, *z*+2/3; (xxiv) −*x*+*y*+1/3, −*x*+2/3, *z*+2/3; (xxv) −*x*, −*y*, −*z*+1; (xxvi) *y*+1/3, −*x*+*y*+2/3, −*z*+2/3; (xxvii) −*x*+*y*−1/3, −*x*+1/3, *z*+1/3; (xxviii) *y*−2/3, −*x*+*y*−1/3, −*z*+2/3; (xxix) *x*−1/3, *y*−2/3, *z*+1/3; (xxx) *x*−*y*+1/3, *x*−1/3, −*z*+2/3; (xxxi) *y*−1/3, −*x*+*y*+1/3, −*z*+1/3; (xxxii) −*y*+1/3, *x*−*y*+2/3, *z*−1/3; (xxxiii) −*y*, −*x*, *z*; (xxxiv) −*x*+*y*+1/3, *y*−1/3, *z*−1/3; (xxxv) *x*+1/3, *y*−1/3, *z*−1/3.
